# Highly uniform hole spacing micro brushes based on aligned carbon nanotube arrays

**DOI:** 10.1186/1556-276X-8-501

**Published:** 2013-11-25

**Authors:** Zhi Yang, Xingzhong Zhu, Xiaolu Huang, Yingwu Cheng, Yun Liu, Huijuan Geng, Yue Wu, Yanjie Su, Hao Wei, Yafei Zhang

**Affiliations:** 1 Key Laboratory for Thin Film and Microfabrication of Ministry of Education, Research Institute of Micro/Nano Science and Technology, Shanghai Jiao Tong University, Shanghai 200240, People's Republic of China; 2 School of Materials Science and Engineering, Shanghai Jiao Tong University, Shanghai 200240, People's Republic of China

**Keywords:** Micro brushes, Carbon nanotube arrays, Chemical vapor deposition

## Abstract

Highly uniform hole spacing micro brushes were fabricated based on aligned carbon nanotube (CNT) arrays synthesized by chemical vapor deposition method with the assistance of anodic aluminum oxide (AAO) template. Different micro brushes from CNT arrays were constructed on silicon, glass, and polyimide substrates, respectively. The micro brushes had highly uniform hole spacing originating from the regularly periodic pore structure of AAO template. The CNT arrays, serving as bristles, were firmly grafted on the substrates. The brushes can easily clean particles with scale of micrometer on the surface of silicon wafer and from the narrow spaces between the electrodes in a series of cleaning experiments. The results show the potential application of the CNT micro brushes as a cleaning tool in microelectronics manufacture field.

## Background

Carbon nanotubes (CNTs) [1,2], a typical one-dimensional nanostructure, have attracted great attention due to their unique combination of electronic, mechanical, chemical, and thermal properties [3-8]. In recent years, CNTs can be prepared mainly by arc discharge [9,10], laser evaporation [11], and chemical vapor deposition (CVD) [12,13]. Due to their mature preparation methods and outstanding properties, CNTs have been extensively exploited in a range of potential applications including nanodevices [14], sensor [15], field emission [16,17], battery [18], and hydrogen storage [19].

The properties of CNTs can be highly enhanced when they are assembled into arrays, which can gain more applications in carbon nanotube devices and further strengthen the advantage of electronic nanodevices [20-23]. Although some material have been successfully aligned [24], it is very difficult to manipulate CNTs to form arrays, which makes it difficult to be economical and practical. Researchers have tried to realize the self-assembly growth of CNT arrays with the help of other auxiliaries [25,26], among which anodic aluminum oxide (AAO) template is one of the important substrates for the growth of CNT arrays. Due to the uniform of the height and the nature, CNT arrays have great potential applications in many fields [25,26].

Brushes are common tools for use in industry and our daily life. Typical materials for constructing brush bristles include animal hairs, synthetic polymer fibers, and metal wires. The performance of these bristles has been limited by the oxidation and degradation of metal wires, poor strength of natural hairs, and low thermal stability of synthetic fibers. CNT is one of the ideal materials for preparing micro brushes, owing to its small size, low density, high thermal stability, outstanding pressure-resistant elasticity, chemically inert, and excellent thermal conductivity properties. Micro brushes based on CNTs can be applied in many fields, such as the cleaning of the nanoscale particles on the integrated circuit, nanofilter to clean air and water and to kill bacteria, and selective adsorption to remove the organic matter and heavy metal ions in solution and the environment [27-29].

In the previous report [27], the hole spacing between the brush bristles was very hard to control. The CNT bristles were easy to take off from the substrate. The above-mentioned disadvantages have hindered their further potential applications. Here, we report a kind of micro brushes based on CNT arrays with the help of AAO template. Because of the regularly periodic pore structure of AAO template, the micro brushes have highly uniform hole spacing. The bristles, CNT arrays, are firmly grafted on the substrates. Finally the cleaning experiments are carried out to evaluate the performance of micro brushes.

## Methods

### Preparation of CNT arrays

At first, a quartz boat and the AAO template were sent into the CVD furnace and the system pressure was pumped to 1 × 10^−2^ Pa. Then, the temperature was raised to 500°C with the introduction of argon gas. After the temperature reaches 500°C, the furnace chamber pressure was controlled at 4,000 Pa for 1 h. Further, the chamber was heated to 700°C and 20 sccm of acetylene was introduced to the system, CNTs grew up in the hole of the AAO template. The reaction time was determined by the thickness of the AAO template. Typically, when the AAO template was 50 mm, the growth time was 2 h. Finally, the system was cooled down in a mixed gas atmosphere of argon and hydrogen. The samples were taken out until the CVD furnace was cooled below 300°C.

### Preparation of micro brushes

The CNT arrays in AAO template were combined on silicon, glass, and polyimide substrates with the assistant of epoxy resin as the adhesive, respectively. The curing temperature was set at 50°C to 80°C for several hours. The samples were soaked into 2 M NaOH in order to completely remove AAO template framework and then washed by deionized water. The micro brushes were prepared after drying.

### The cleaning experiments

Three types of cleaning experiments of particles on the silicon wafer and from the narrow spaces between the electrodes with the distance of 2 and 100 μm were carried out, respectively. The mixed particles are the silica with the diameter of 1 μm and epoxy resin powder with the diameter of 3 to 5 μm, including inorganic and organic particles. They were spilled on the surface of the substrate, the as-prepared micro brushes were used to clean for several times.

### Characterizations

Transmission electron microscope (TEM) images were taken using a JEOL JEM-2100 microscope (JEOL, Akishima-shi, Tokyo, Japan) operating at 200 kV. The morphology of the samples was observed by scanning electron microscopy (SEM) using a Carl Zeiss (ULTRA 55, Carl Zeiss, Oberkochen, Germany) with energy dispersive X-ray (EDX, INCA PentaFET × 3, Model: 7426, Oxford Instruments, Abingdon, Oxfordshire, UK) spectrometry mode. The Raman spectra were obtained using a Senterra R200-L Raman spectrometer (Bruker, Germany) with a 514-nm line of laser source.

## Results and discussion

**Figure 1:**
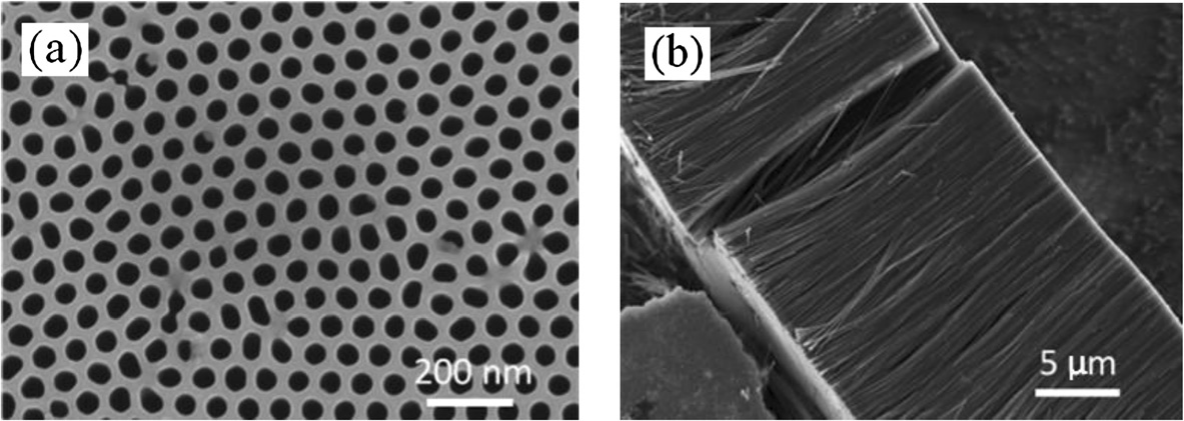
**SEM images of the samples. (a)** AAO template and **(b)** CNT arrays.

To get the morphology, composition and the degree of graphitization of CNT arrays, the resultant SEM, TEM, EDX, and Raman spectra were used for characterization. As shown in Figure 1a, the AAO template has flat surface with the regularly periodic pore structure. After completely removing AAO template framework, the resultant CNT arrays were obtained as shown in Figure 1b. The aligned CNTs have high density in consistent with that of the template.

**Figure 2:**
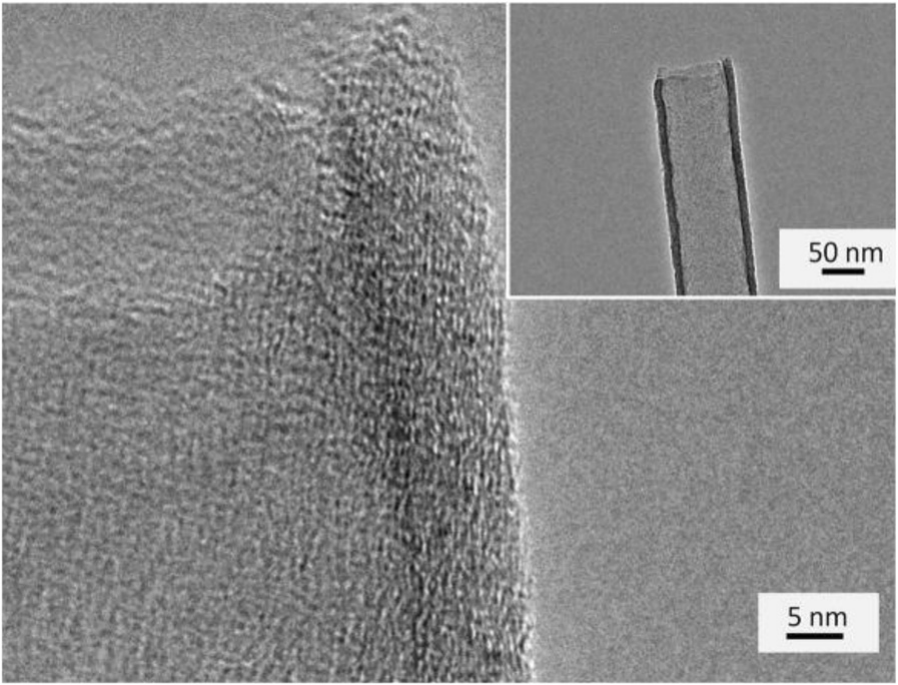
**TEM images of CNT.** The inset is the low magnification image.

Figure 2 is TEM image of CNT arrays after ultrasonic dispersion. It can be observed that CNTs with the assistance of the AAO template have good opening channels with the thickness of CNT walls of 8 to 10 nm, including about 25 layers. So CNTs prepared in our experiment are multi-walled ones. Compared with other reported research results [13], the obtained CNTs have clean and smooth surface with high degree of graphitization.

**Figure 3:**
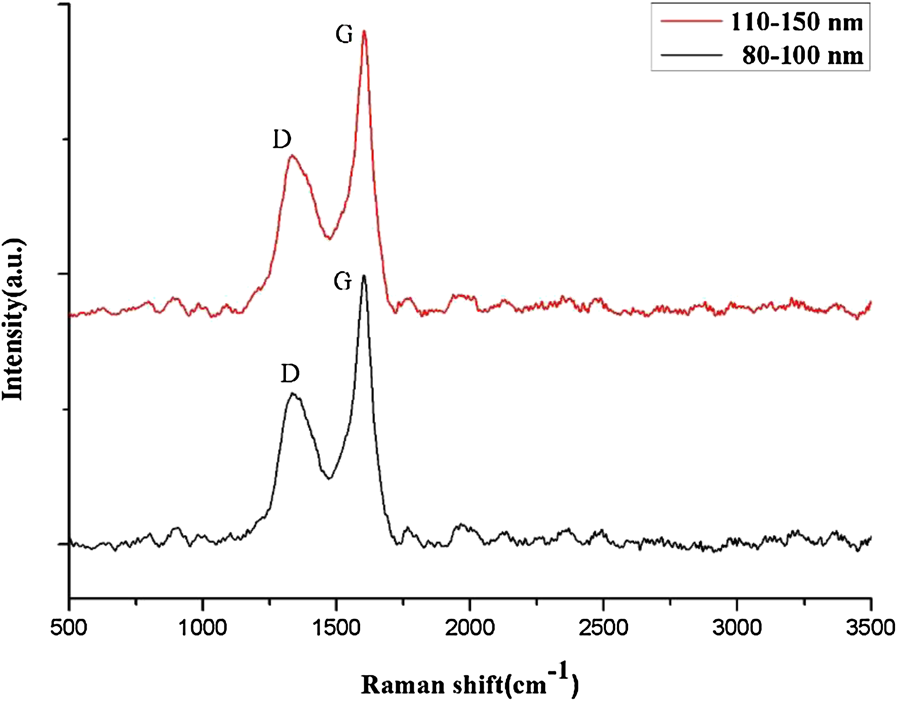
Raman spectra of CNT arrays.

Figure 3 presents the Raman spectra of CNT arrays with two kinds of diameters (80 to 100 and 110 to 150 nm). It is noted that there are two obvious peaks in the 1,350 and 1,580 cm^−1^, which are the D and G peak, respectively. By comparing the intensities of two peaks, the *I*_G_/*I*_D_ of CNTs is about 2, which is better than those of other works using the same method [30].

In general, the diameter of CNTs is in consistent with pore size of AAO template. The roughness of CNTs has great relation with that of the hole wall of AAO template. In previously reported CVD experiments [12], the temperature of the system was increased quickly to reaction temperature and then immediately started the CVD experiment. In this process, the temperature directly rose from room temperature to reaction temperature; in other words, the sample has always been in a rapid heat treatment condition. Part of the internal thermal stress of the template was released through high-temperature deformation, but the majority of the thermal stress could not get released due to the rapid heating process. Thermal annealing is an effective method in thermal stress release [31]. In order to improve graphitization degree of CNTs, a heat preservation pretreatment for 1 h under 500°C was added during the fast heating process so that the template could be fully stretched and the deformation stress will be released completely.

**Figure 4:**
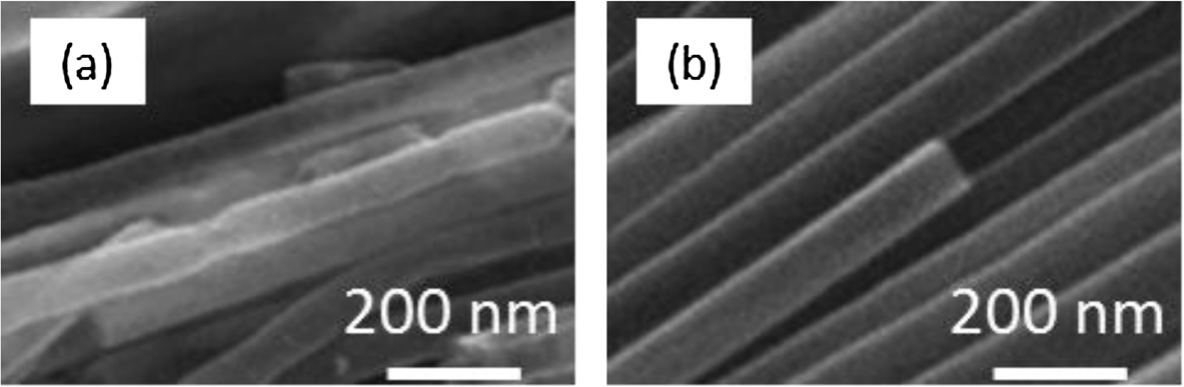
**SEM images of CNTs. (a)** Without and **(b)** with thermal insulation pretreatment.

Figure 4 is SEM images of CNT arrays with and without pretreatment, respectively. In Figure 4a, it can be observed that the lengths of the CNTs are inhomogenous and the walls are rough without pretreatment. Figure 4b clearly shows the morphology of CNT arrays with pretreatment. Compared with that of Figure 4a, the lengths of CNTs are perfectly uniform and aligned with a great enhancement of graphitization degree with pretreatment. The brushes based on the CNT arrays with the heat preservation pretreatment may clean the particles better than those without the pretreatment due to their flexibility and recoverability. The reason why heat preservation has so strong effect is that it can change the inner stress distribution of AAO template, thus affect the hole roughness of the AAO template.

**Figure 5:**
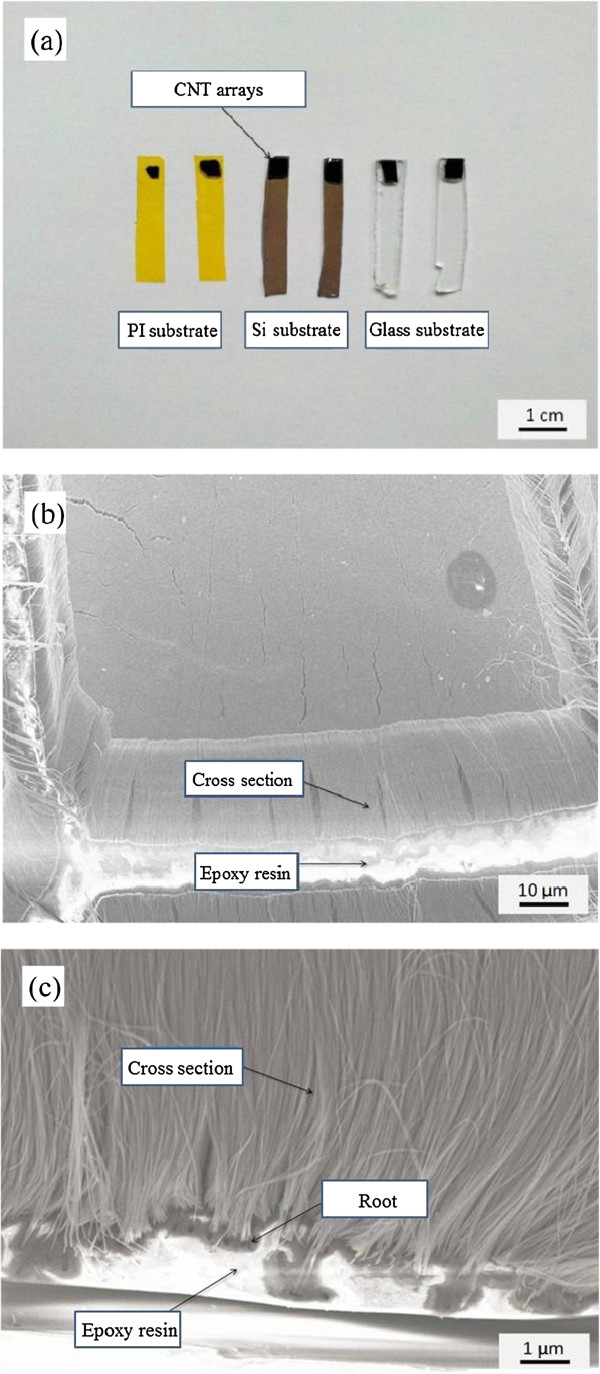
**Photo and SEM images of micro brush. (a)** Photo of micro brushes, **(b)** low magnification SEM image of micro brush, and **(c)** high-magnification SEM image of micro brush.

**Figure 6:**
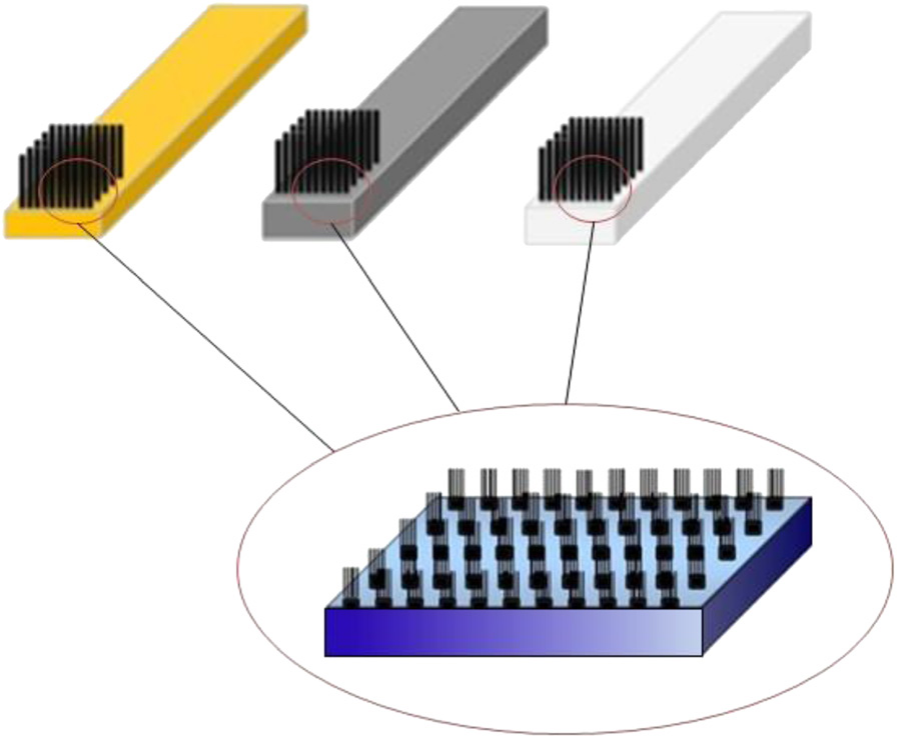
Schematic diagram of micro brush.

Epoxy resin was adopted as the adhesive of bristles and substrate, because it can avoid corrosion in acid, alkali, and high-temperature atmosphere. In practical applications, brush should combine with different substrates to meet multiple requirements, such as electrical conductivity, survivability, and mechanical properties. So different micro brushes from the CNT arrays were constructed on the substrate of silicon wafer, glass sheet, and polyimide, respectively. In Figure 5a, we can observe that the three micro brushes have toothbrush-like structures, which enable them to meet different requirements and environments. It is shown that the bristles of micro brush have a fairly uniform height. If the bristles and substrate combine loosely, the external force in practice will lead to severe shedding of bristles which will reduce the lifetime of use. The adhesive degree of bristles and substrate is showed in Figure 5c. The upper part shows the uniform CNT arrays, namely the bristles. It can be clearly seen that the bristles are firmly embedded in epoxy resin and closely combined with the substrate, which is of great benefit to the use lifetime of micro brushes. The schematic diagram of micro brush is showed in Figure 6.

**Figure 7:**
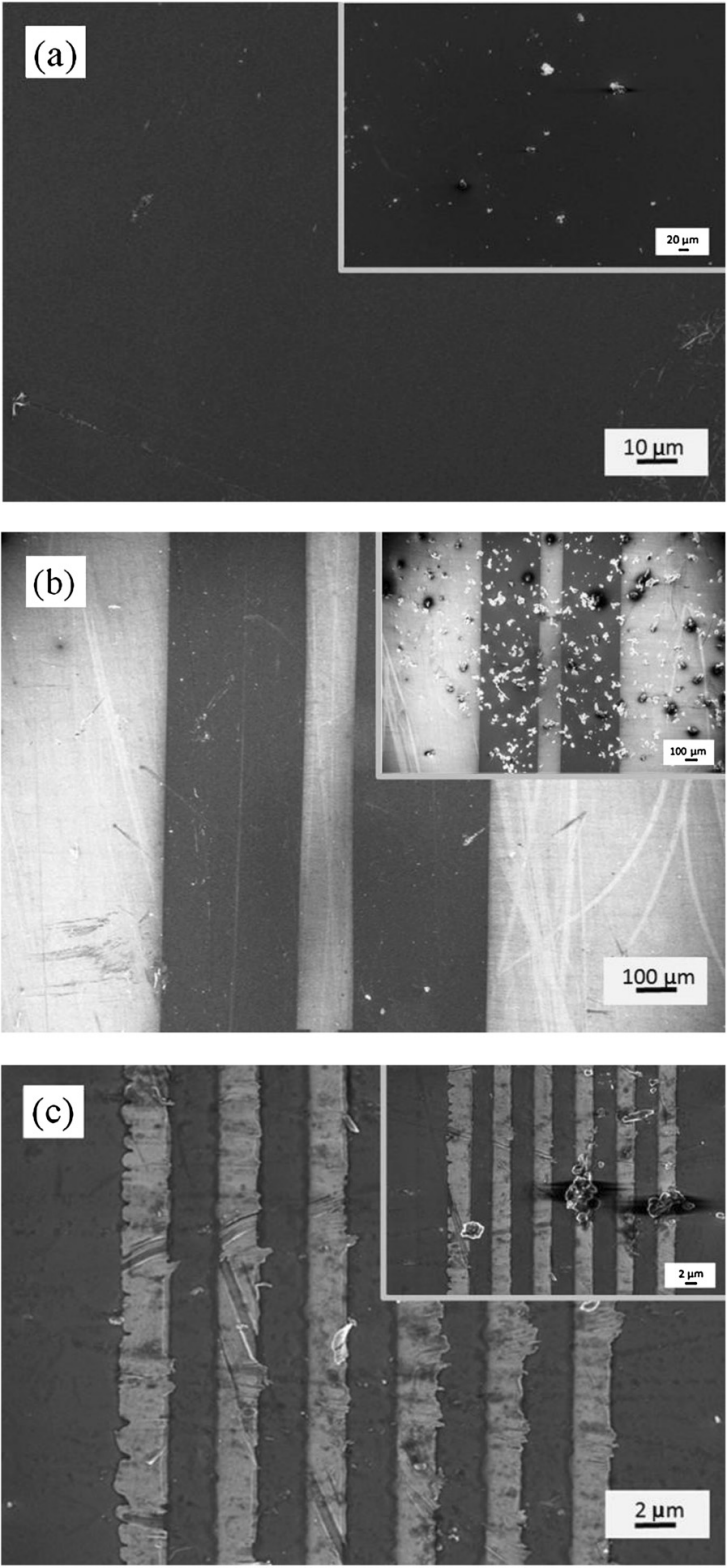
**The cleaning experiments of micro brush.** The surface of **(a)** silicon wafer, **(b)** the electrode with gap of 100 μm, and **(c)** the electrode with gap of 2 μm.

The research of micro brushes in cleaning the particles in the smooth plane and narrow space will be very meaningful. Figure 7 shows SEM images of the substrate before and after the brush cleaning. In Figure 7a, the particles are found to be almost cleaned from the surface of silicon wafer. The micro brushes were further used to clean rough surfaces, for example, narrow space between the electrode with the width of 100 and 2 μm, as shown in Figure 7b,c. This was done by sweeping along the narrow space direction several times, which removed nearly all of the particles on the electrodes and between the ones, indicating that the flexible CNT array bristles can adapt to the geometry of narrow spaces. In Figure 7c, some tiny particles still remain on the surface, due to smaller space between the electrodes.

## Conclusions

In summary, we have demonstrated that micro brushes based on CNT arrays were successfully fabricated. Firstly, the preparation of CNT arrays by a CVD method in AAO template was studied. The results show that the quality and degree of graphitization of CNT arrays can be improved significantly through a heat preservation pretreatment method. Secondly, three types of micro brushes were obtained on silicon, glass, and polyimide substrates with the assistance of epoxy resin, respectively. The hole spacing of the micro brushes is highly uniform owing to the regularly periodic pore structure of AAO template. The CNT arrays were firmly grafted on the substrates as bristles. The cleaning experimental results show that the particles on the surface of silicon wafer and between the electrodes can almost be swept away. The results expand the cleaning practicality of micro brushes in microelectronics manufacture field.

## Competing interests

The authors declare that they have no competing interests.

## Authors' contributions

ZY carried out the sample preparation, participated on its analysis, performed all the analyses except TEM and Raman analyses, and wrote the paper. XZZ, XLH, and YWC also wrote the paper and analyzed the samples. YL performed the TEM analysis. HJG and YW participated on the Raman analysis and proof corrections. YJS, HW, and YFZ participated in the study guidance and paper correction. XH has read and approved this manuscript. All authors read and approved the final manuscript.
